# Changes in *Emberiza* bunting communities and populations spanning 100 years in Korea

**DOI:** 10.1371/journal.pone.0233121

**Published:** 2020-05-27

**Authors:** Chang-Yong Choi, Hyun-Young Nam, Han-Kyu Kim, Se-Young Park, Jong-Gil Park

**Affiliations:** 1 Department of Forest Sciences, Seoul National University, Seoul, South Korea; 2 Research Institute of Agriculture and Life Sciences, Seoul National University, Seoul, South Korea; 3 School of Biological Sciences, Seoul National University, Seoul, South Korea; 4 Korea National Park Service, Wonju, Gangwon Province, South Korea; Nanjing Forestry University, CHINA

## Abstract

The rapid decline of a few *Emberiza* bunting species is increasing conservation concerns, especially in Asia. However, temporal changes in communities and populations of buntings, ones of the most common migratory songbirds in Korea, have not been quantitatively assessed. To understand how the status of buntings has changed over the past 100 years, we collated abundance data from museum collections and bird-banding records between 1910 and 2019. We also used presence–absence data for buntings collected by a nationwide census scheme between 1997 to 2012. Our analysis showed that bunting communities reconstructed from museum-specimen and bird-banding data were not significantly different; however, community composition differed over time. The Meadow (*E. cioides*), Yellow-throated (*E. elegans*), Black-faced (*E. spodocephala*), Rustic (*E. rustica*) and Chestnut Buntings (*E. rutila*), which are still common or were once common species, significantly affected the temporal changes in bunting community composition. There were no recent changes in the presence of Rustic and Chestnut Buntings since 1997, but they caused medium-term changes in the bunting community composition, suggesting that there was a sharp to moderate decline in their numbers in the past. The probability of the presence of six bunting species decreased annually, with the most prominent decline in two common breeders, the Meadow (-2.99%/year) and Yellow-throated Buntings (-1.82%/year). This finding suggests that breeding buntings in Korea are under high pressure, as are the migratory buntings. Moreover, despite its recent population decline, the Yellow-throated Bunting was still a major contributor to the community, suggesting that bunting diversity has also been deteriorating while bunting populations are shrinking. Long-term monitoring schemes across their distribution ranges, international cooperation for identifying major threats and key areas of conservation, and law enforcement against illegal hunting and habitat loss are strongly required to mitigate the on-going decline of buntings in Korea and Asia.

## Introduction

The status of the world’s birds has been in a steady and continuing trend of deterioration, and diverse anthropogenic threats are the main drivers of this decline [1–4]. A recent integrated study found that there has been a loss of around 3 billion the North American birds over the past 48 years, including once-common species, and there are now 29% of the 1970 levels [5]. An analysis of the International Union for Conservation (IUCN) Red List showed that Asian songbirds are less threatened overall than other bird groups [3], but they have received little scientific or conservation attention [6] and are currently declining at an alarming rate [3].

Populations of some migratory songbirds wintering in southeast Asia are known to have declined, and the loss of their habitats is suspected to be the main driver of this decrease [7, 8]. However, many migratory bird populations in temperate Asia have also declined without a clear loss of habitat in their breeding grounds [7, 9]. For example, the number of Yellow-breasted Buntings (*Emberiza aureola*) and other migratory grassland birds that breed in Hokkaido, Japan has declined, although their breeding habitats have been nearly unchanged [9]. Kamp [10] identified that the Yellow-breasted Buntings have been significantly threatened by overexploitation for human consumption in their non-breeding grounds or migratory routes. The negative consequences of habitat loss and wildlife overexploitation are often cumulative [11], and it is clear that hunting is still a major threat in this region [7, 10]. However, threats to songbirds are less studied, assessed, and therefore understood in Asia [7], and little information is available on the changes to the communities and populations of Asian songbirds. This knowledge gap on the songbirds and threats is due to the diversity and complexity of habitat change patterns, migratory routes crossing jurisdictional boundaries and connecting different habitats in distant geographic regions, and, differently from waterbirds, the lack of systematic and consistent monitoring schemes in Asia [12].

**Fig 1:**
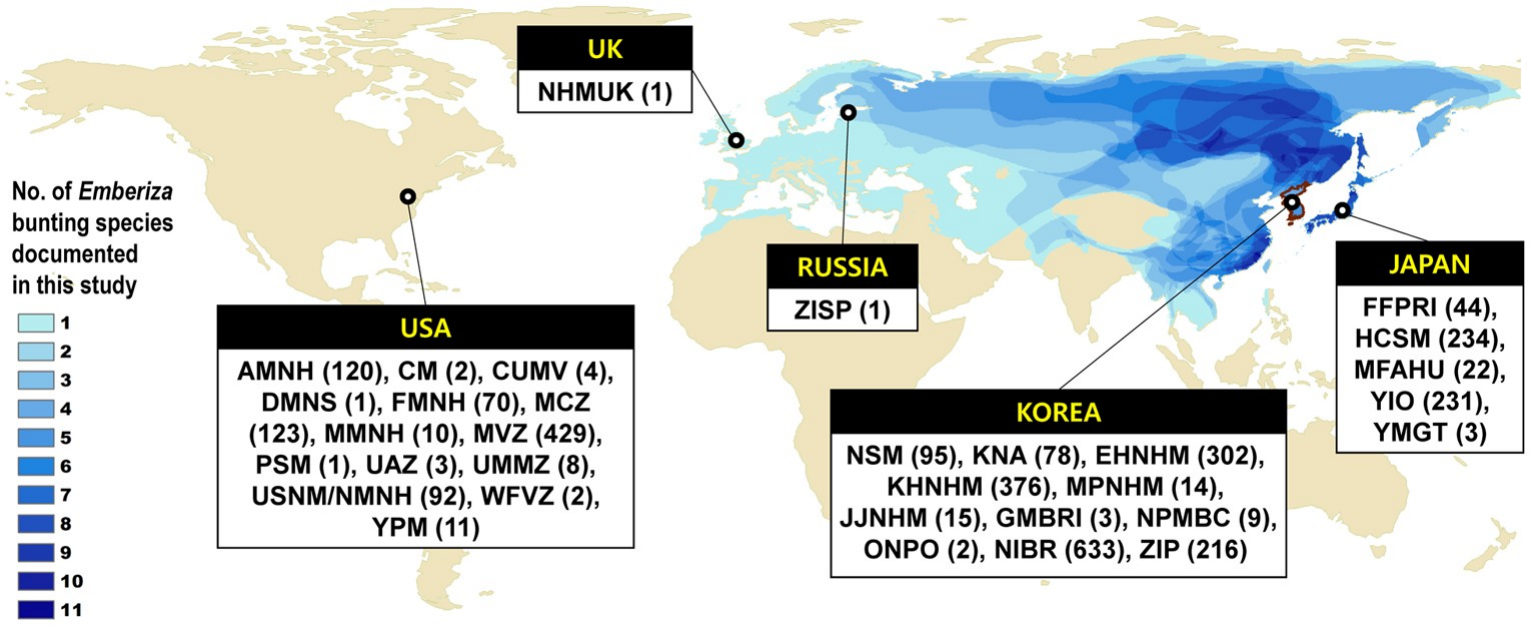
Overlapped distribution ranges of 17 *Emberiza* species recorded in Korea and used in this study. Overlapped distribution ranges are marked in blue contours. The museums and institutions that have preserved *Emberiza* bunting specimens collected in Korea since 1910 were abbreviated and have been marked on the countries in which they operate. The number of specimens used in this study were noted in parentheses. The base map was made with Natural Earth, and the distribution ranges were from the IUCN Red List for birds prepared by BirdLife International.

Buntings in the genus *Emberiza* are small-sized songbirds of the Palearctic region [13], and most of the buntings in the northern latitudes are migratory species that depend on many geographic areas and habitats along their migration routes. For instance, the Rustic Bunting (*E. rustica*), which breeds in the Eurasian taiga from Fennoscandia in the west to the Kamchatka Peninsula in the east, migrates to small geographic areas in East Asia, such as China, Japan, and Korea [13–15]. Buntings are one of the most important songbirds in Korea, as seen by the fact that more than 64% of the total birds captured in mist nests in Korea between 1964 and 1970 were buntings [16, 17]. However, recent studies indicate that the once common, abundant and widespread buntings are rapidly declining; populations of the Yellow-breasted [9, 10] and Rustic Buntings [15] seem to have collapsed due to unsustainable trapping for food and ritual release in Southeast Asia [7, 9, 10, 15, 18]. Despite the rapid and remarkable population decline in some buntings, many of the 22 *Emberiza* species reported in Korea are still regarded as common and abundant [19]. Nevertheless, their status has never been assessed in Korea, which lies in the middle of the East Asian Flyway, one of the main migration routes of Asian songbirds [7] (Fig 1).

Bird banding records may provide reliable sources to monitor the change in bird populations and communities when trapping efforts are quantified and accounted for [20, 21], but such quantitative data are not available before the 1960s, when bird banding first started in the Republic of Korea (South Korea). Alternatively, there are available museum specimens collected both in South Korea and in the Democratic People’s Republic of Korea (North Korea) since the 1900s. These specimens may also provide clues on bird populations or communities; however, they are uncommon and are present in many different facilities. Museum specimens have been used for retrogressive studies in many biodiversity studies [22, 23], but trials that have used these two methods to detect any changes in bird populations and communities over a long-term period are still lacking. In addition, there is no other remaining source that can be used to assess how the species compositions and the numbers of Korean birds, including buntings, have changed over the past century. This study aimed to understand the short-, medium-, and long-term changes in communities and populations of *Emberiza* buntings over the past 100 years in Korea. Because there is no systematic information on their status over time, we collated available abundance data from museum collections and bird banding records between 1910 and 2019 to reconstruct the communities of the buntings in three different periods. Therefore, recognizing the need for quantitative assessment of their status, we tested (1) whether the bunting communities reconstructed from data collected using two alternative methods were different, (2) whether the reconstructed bunting communities have changed over time, and (3) which species mainly contributed to the change in bunting communities over time. Given the known rapid decline in some buntings in Asia and Europe [7, 9, 15], we also used presence–absence data for buntings collected through a nationwide census scheme between 1997 to 2012 to identify (4) possible short-term population changes in major bunting species in Korea.

## Materials and methods

### Specimen data for bunting community analysis

To examine bunting specimens collected in Korea, we accessed the Korea Natural History Research Information System (NARIS; https://www.naris.go.kr) and searched for collection records of any bunting species deposited in South Korea. The NARIS is a national database program managed by the National Science Museum (NSM) with support from the Korean Ministry of Science and Information and Communication Technology. The search results included specimens preserved in NSM, Korea National Arboretum (KNA), Ewha Womans University Natural History Museum (EHNHM), Kyunghee University Natural History Museum (KHNHM), Mokpo Natural History Museum (MPNHM), Folklore and Natural Museum of Jeju Special Self-Governing Province (JJNHM), Gunsan Migratory Bird Research Institute (GMBRI), and Gyeryongsan Natural History Museum (GNHM) that hold bunting specimens. We also visited NSM, KNA, EHNHM, KHNHM, and Hannam University Natural History Museum (HUNHM) from 2007 to 2009 to examine specimens found or missing from searches. Catalogs of bird specimens in the Korea National Park Service were also examined in 2010, and some positively-confirmed results from the Migratory Bird Center of the Korea National Park Research Institute (NPMBC) and Odaesan National Park Office (ONPO) were included. Recent bird specimens and collected samples from the National Institute of Biological Resources (NIBR) were independently searched through its own database system (https://species.nibr.go.kr) (Fig 1). Bunting specimens deposited in North Korea were examined and reviewed by Tomek [24]. From the literature, we collated specimen data that had known collection dates that were deposited in the Zoological Institute of the Korean Academy of Sciences in Pyongyang (ZIP), North Korea [24] (Fig 1).

Since many specimens had been exported to and preserved in foreign museums and institutions, we also searched for Korean buntings in the VertNet (http://www.vertnet.org), an integrated online data portal for vertebrate specimens and in available catalogs and databases on historical bird collections in Korea. We identified bunting specimens in the American Museum of Natural History (AMNH), Carnegie Museum of Natural History (CM), Cornell University Museum of Vertebrates (CUMV), Denver Museum of Nature & Science (DMNS), Field Museum of Natural History (FMNH), Harvard University Museum of Comparative Zoology (MCZ), Bell Museum of Natural History (MMNH), Museum of Vertebrate Zoology of UC Berkeley (MVZ), Museum of Natural History of the Smithsonian Institution (USNM/NMNH), James R. Slater Museum of Natural History (PSM), University of Arizona Museum of Natural History (UAZ), National University of Michigan Museum of Zoology (UMMZ), Western Foundation of Vertebrate Zoology (WFVZ), and the Vertebrate Zoology Division of Yale Peabody Museum (YPM).

For collections preserved in Japan, specimens in the Yamashina Institute for Ornithology (YIO) of Japan were checked first through its database on specimens and their images (https://decochan.net). Based on the published literature and catalogs, a few specimens in the following Japanese facilities were also searched and included: the Forestry and Forest Products Research Institute (FFPRI) [25, 26], Museum of Agriculture Hokkaido University (MFAHU) [27], Yamagata Prefectural Museum (YMGT) [28], and Himeji City Science Museum (HCSM) [29]. In addition, one Yellow-throated Bunting (*E. elegans*) specimen in the Natural History Museum’s database (NHMUK; https://data.nhm.ac.uk) in the UK and one Meadow Bunting (*E. cioides*) specimen in the Zoological Institute of the Russian Academy of Sciences in Saint Petersburg (ZISP), that was examined by Tomek [24], were also included (Fig 1).

We only included specimen records with confirmed collection dates between 1910 and 2019. Specimens identified from South and North Korea, Japan, the UK, and the US were grouped into three different periods: Period I (1910–1949), Period II (1950–1989), and Period III (1990–2019). Most bunting specimens collected in Period I are deposited in Japan while those in Period II are in the US; most bunting specimens collected in Period III were deposited in Korea. We treated the number of specimens with a known collection date as the possible abundance of each species at a given period.

To assess the reliability of the data sources, we also closely examined actual specimens and their image sources of more than 450 specimens deposited in South Korea and Japan and 24 available specimens in the National Museum of Natural History (NMNH). We found that only one specimen was different from the catalog in a Korean museum and that the albino Meadow Bunting (*Emberiza cioides*) was misidentified as a Snow Bunting (*Plectrophenax nivalis*); it was not included in this study as its collection information was not complete. According to this preliminary assessment, we used collection data from catalogs and databases because possible errors in the catalogs and databases may not cause any significantly changes in the following analyses and results.

### Bird-banding data for bunting community analysis

The Migratory Animal Pathological Survey (MAPS) was the most extensive and systematic survey of birds ever conducted in East and Southeast Asia, capturing and banding a total of 1,165,288 birds of 1,218 species between 1962 and 1971 [16, 17]. According to the MAPS, more than 186,000 birds were marked with metal bands in Korea between 1964 and 1970 [16, 17]. This information provides invaluable baseline data to understand the relative abundances of buntings in Period II after the Korean War (1950-1953).

Systematic bird-banding surveys for forest songbirds in Korea were nearly discontinued after the end of the MAPS, but they resumed in 1993 and are currently managed by NIBR [30]. We used the national bird banding data collected from 1993 to 2017 [30] to identify the number of banded buntings by species. In addition to the national banding data, we also included the authors’ private banding records of buntings from 2017 to 2019. The number of buntings was considered representative of the general abundance of buntings in the bunting community in Period III.

### Changes in bunting communities

Dissimilarity is an index commonly used in ecology to quantify the compositional difference between two communities [31–33]. To quantify dissimilarities between the bunting communities assessed by the two methods in the different periods, we first calculated the Bray–Curtis dissimilarity matrix based on species abundances as defined in Koleff et al. [31]. We then ran the permutational multivariate analysis of variance (PERMANOVA) using the distance matrices [33, 34] to test for differences and estimate components of variation by sampling method (specimen collection vs. bird banding) and period (Periods I, II, and III). The PERMANOVA is a permutation test with pseudo-F ratios for partitioning distance matrices among sources of variation and fitting linear models to distance matrices [33–35]. It tests whether the centroids of the groups, as defined in the space of the chosen resemblance measure, are equivalent for all groups [35].

The similarity percentage, also known as SIMPER, indicates the average contribution of each species to the average overall Bray–Curtis dissimilarity [33, 34, 36]. Because a species with a consistently high contribution to the dissimilarity between groups is a good discriminating species [32], a SIMPER function performs pairwise comparisons of sample groups [33, 34] and identifies the species that are most responsible for the dissimilarity patterns observed between the sample groups compared by period [32]. The contribution of individual species *i* to the overall Bray–Curtis dissimilarity *d*_*jk*_ is given by Eq (1):

$$\begin{array}{c} d_{ijk}=\frac{\left|x_{ij}-x_{ik}\right|}{\sum_{i=1}^{S}(x_{ij}+x_{ik})} \end{array}$$ (1)

where *x* is the abundance of species *i* in sampling units *j* and *k* [33]. The overall index is the sum of the individual contributions of all *S* species [33] given Eq (2):

$$\begin{array}{c} d_{jk}=\sum_{i=1}^{S}d_{ijk} \end{array}$$ (2)

For multivariate ordination analysis of the bunting communities, we used non-metric multidimensional scaling (nMDS) based on Bray–Curtis dissimilarity [32–34]. Then we visualized a plot of species scores, group scores, and variables (methods and periods) using two dimension axes. The *vegan* package [34] was used in the R 3.5.1 environment [37] for the PERMANOVA, SIMPER, and nMDS analyses.

### Presence–absence data for bunting population analysis

The Nationwide Natural Environment Survey (NNES), required by the Natural Environment Conservation Act of South Korea, is a national monitoring scheme of natural resources including birds [38]. The NNES has covered all of the South Korean territory since 1986 in phases that are commonly five-years long [38]. The detailed protocols for bird surveys slightly changed between the phases. We did not use the survey records collected in the first phase (1986-1996) as they had different survey protocols and units, but did use the records collected from the second and third phases, which used presence–absence as the main protocol.

A total of 936 regional units across Korea were surveyed in the second phase (1997–2005), while 1–9 sectional units of 944 reference maps (at a sclae of 1:25,000) were monitored in the third phase (2006–2013) [38]. The sampling area and shape of the regional unit were variable in the second phase because each unit was a watershed-based, but the area of a rectangular sectional unit was about 2 km^2^ in the third phase because each unit was a map-based.

Presence–absence data provide information on whether a species was detected at a set of sampling sites [39]. The presence or absence of a species was determined by seasonal surveys (which occur more than three times a year) between March and December in each survey unit. We accessed the Digital Library of the Korean Ministry of Environment (http://library.me.go.kr) and collated the presence–absence data for 12 bunting species between 1997 and 2012 in a total of 5,826 regional and sectional units in South Korea. Both survey units included diverse types of habitats from lowlands to high mountains in South Korea, and some habitats were favorable to buntings while others were not.

### Changes in bunting populations

The value of presence–absence surveys is often discounted or interpreted informally in studies of population dynamics, but this survey method may sometimes be the most cost-effective and only feasible alternative for monitoring of larger areas [40, 41]. Species detection using this method is often imperfect and leads to false absence records [39]. An occupancy model using presence–absence data includes the probability of species occurrence at a site (*ψ*) and the detection probability of a species at a site where it is present (p*); this model can provide more information on species distribution [39]. However, given that there are no known reliable estimates on the detection probability of buntings and there are inconsistent individual survey efforts across the units in the NNES scheme, logistic regression was a feasible alternative method to analyze presence–absence data [39]. We used the general linear model (GLM) in the *glm2* package [42] of R 3.5.1 [37], which provided predictions including the effects of quantitative variables [43]. We used the quasi-binomial GLM, which was developed to overcome an assumption of the binomial model that individuals are randomly distributed over sample sites, and the logit link function was selected to predict the probability of presence (between 0 and 1) of each bunting species [43]. This analysis provided fundamental information about the probability of presence (measured by observing the species or by its encounter rate) [39]; though this is not the robust probability of species occurrence, it may be a useful index for bunting population changes.

## Results

### Community changes in buntings

We collated available specimen and bird-banding data for 17 bunting species collected in Korea between 1910 and 2019 (Table 1, S1–S3 Tables). We identified 1,412 Korean specimens with known collection dates deposited in foreign facilities, and 1,743 specimens deposited in Korean museums and institutes (S1 Table). Among the 3,155 bunting specimens, 523 were collected in Period I, 1,956 in Period II, and 676 in Period III (Table 1). Based on the abundance of specimens, the most commonly collected species were the Meadow Bunting in Period I, the Yellow-throated, Rustic, Meadow, and Black-faced Buntings (*E. spodocephala*) in Period II, and the Yellow-throated Bunting in Period III (Table 1). The MAPS data indicated that 119,774 buntings were captured and marked with metal bands during Period II, and the Rustic and Chestnut Buntings (*E. rutila*) were the most commonly banded birds (Table 1). However, out of the 29,841 buntings captured during Period III, the Black-faced and Yellow-throated Buntings were the most common species (Table 1, S2 and S3 Tables).

**Table 1:** The number of collected and banded *Emberiza* buntings in three different periods in Korea. There was no bird banding study prior to the 1950s (Period I).

Species	No. of specimens collected			No. of birds banded		
	Period I (1910s-1940s)	Period II (1950s-1980s)	Period III (1990s-2010s)	Period II (1950s-1980s)	Period III (1990s-2010s)
*Emberiza aureola*	27	137	5	266	224
*Emberiza chrysophrys*	9	11	18	18	1,426
*Emberiza cioides*	123	284	17	3,156	73
*Emberiza elegans*	64	355	395	3,074	6,784
*Emberiza fucata*	37	105	5	888	275
*Emberiza jankowskii*	6	0	0	0	0
*Emberiza leucocephalos*	1	11	0	9	6
*Emberiza pallasi*	30	56	9	0	983
*Emberiza pusilla*	11	17	8	57	1,421
*Emberiza rustica*	68	336	21	61,055	2,572
*Emberiza rutila*	22	192	26	46,826	2,422
*Emberiza schoeniclus*	3	22	1	11	265
*Emberiza spodocephala*	87	238	59	1,972	11,092
*Emberiza sulphurata*	0	1	9	11	127
*Emberiza tristrami*	24	166	61	2,079	2,122
*Emberiza variabilis*	0	0	9	0	14
*Emberiza yessoensis*	11	25	33	352	35
**Total**	523	1,956	676	119,774	29,841

The results of the PERMANOVA confirmed that the composition of the bunting community did not differ for the two data-collection methods (specimen vs. bird-banding; F = 2.3316, R^2^ = 0.1679, *P* = 0.208; Table 2). On the other hand, bunting communities were significantly dissimilar between periods (Periods I, II, and III; F = 9.5583, R^2^ = 0.6881, *P* = 0.017; Table 2), indicating that the dissimilarities were a result of temporal change.

**Table 2:** The results of the permutational multivariate analysis of variance using distance matrices of *Emberiza* bunting communities in Korea.

Source	df	Sum Squares	Mean Squares	F	R^2^	*P*
**period**	1	0.0090	0.0090	9.5583	0.6882	0.0167
**method**	1	0.0022	0.0022	2.3316	0.1679	0.2083
**Residuals**	2	0.0019	0.0009		0.1440	
**Total**	4	0.0130			1.0000	

**Fig 2:**
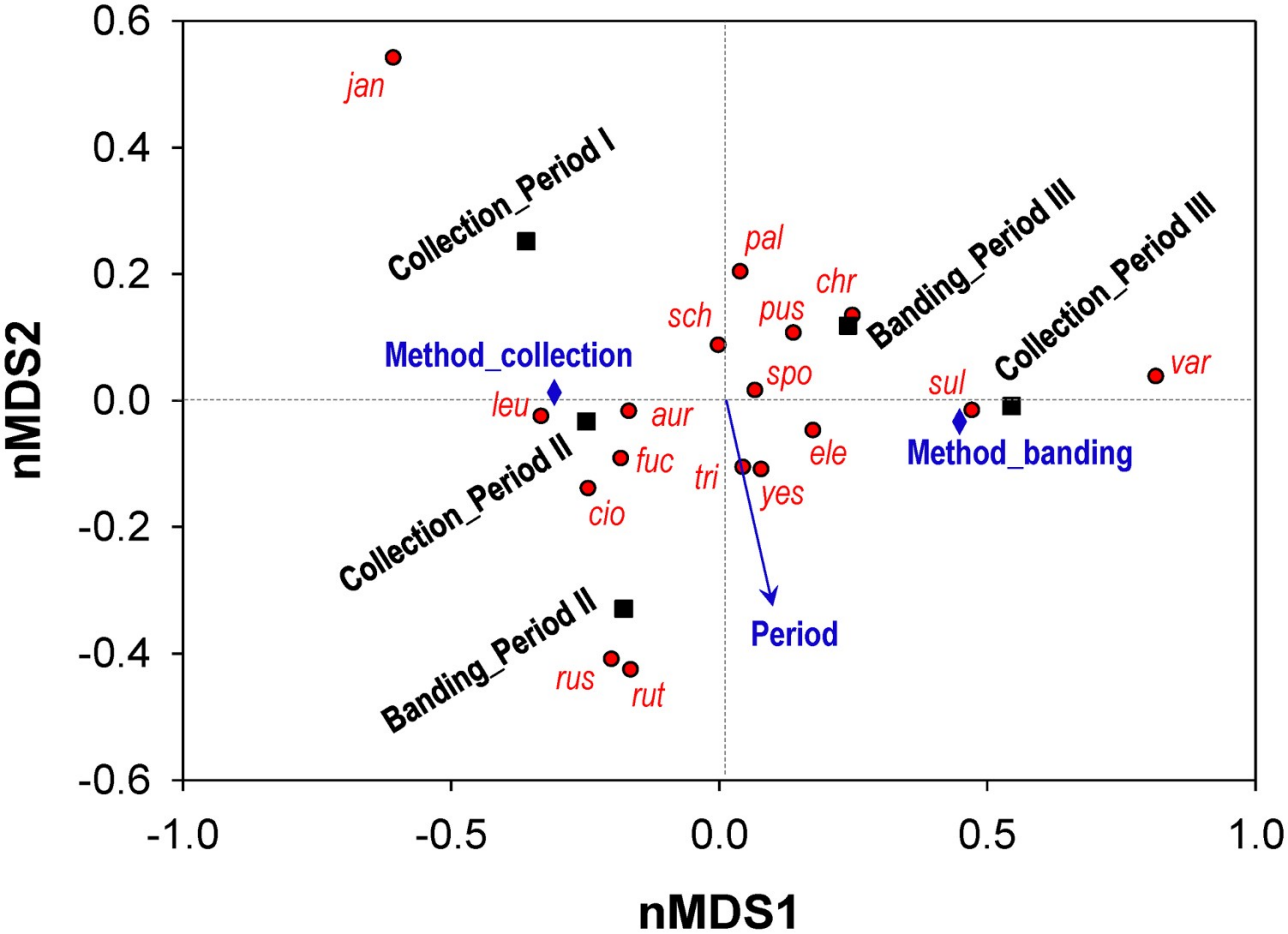
Two-dimensional non-metric Multidimensional Scaling (nMDS) ordination of data samples. Species scores, group scores, and variables (methods and periods) from the nMDS ordination were plotted, and the patterns in bunting community composition were revealed based on the dissimilarities. Each *Emberiza* species was marked by the first three letters of its epithet in a lower case.

The ordination through the nMDS revealed a cloud of species according to the period and the methods (Fig 2). The first dimension has positive associations with the period and the samples from bird banding data, but negative associations with the samples from museum collections. Periods had large negative loading on the second dimension, while survey methods did not. The Jankowski’s (*E. jankowskii*) and Grey Buntings (*E. variabilis*), only collected in Period I and only banded in Period III respectively, were distinct species from the others forming a broad central cloud on the ordination plot. The Rustic and Chestnut Buntings that were dominantly recorded by bird banding in Period II also formed a distinct group on the plot.

The pairwise comparison of bunting communities demonstrated that six species were distinctive in each pair-wise comparison between periods and that they contributed to 83-90% of the total changes in the communities (Table 3). In particular, the Yellow-throated and Black-faced Buntings contributed to long-term changes in the bunting communities (Period I vs. III), whereas the Rustic and Chestnut Buntings mainly contributed to medium-term changes (Period II vs. III) (Table 4). The relative abundances of the Yellow-throated and Black-faced Buntings increased throughout the periods, but those of the Rustic and Chestnut Buntings decreased especially between Period II and III (Table 3).

**Table 3:** The pairwise comparison of *Emberiza* bunting communities from the combined museum collection and bird banding data in Korea. Average abundance (%) in each period, average contribution to overall Bray–Curtis dissimilarity (%) with its standard deviation, and ordered cumulative contributions of six top species were given. The average contribution indicates the species contribution to average between-group dissimilarity. Cumulative contributions are based on the average contributions, but they are standardized to sum up to total 100.

Species	Abundance (%)		Average contribution (%)	Cumulative contribution (%)
	Early	Later		
**Period I vs. III**				
*Emberiza elegans*	12.38	23.52	24.87 ± 3.87	32.22
*Emberiza spodocephala*	16.83	36.54	19.29 ± 23.98	57.20
*Emberiza rustica*	13.15	8.50	6.08 ± 3.06	65.08
*Emberiza tristrami*	4.64	7.15	5.00 ± 2.70	71.56
*Emberiza cioides*	23.79	0.29	4.50 ± 6.13	77.39
*Emberiza rutila*	4.26	8.02	4.12 ± 5.35	82.73
**Period II vs. III**				
*Emberiza rustica*	50.43	8.50	27.19 ± 21.06	33.47
*Emberiza rutila*	38.62	8.02	20.46 ± 16.38	58.67
*Emberiza spodocephala*	1.82	36.54	12.16 ± 14.83	73.63
*Emberiza elegans*	2.82	23.52	6.61 ± 9.08	81.77
*Emberiza cioides*	2.83	0.29	3.87 ± 4.26	86.53
*Emberiza tristrami*	1.84	7.15	2.96 ± 2.68	90.18

**Table 4:** Six main *Emberiza* species have caused changes in bunting communities and populations in Korea over time. The International Union for Conservation of Nature (IUCN) status in parentheses indicates the Red List Category and its population trend as of March 2020 (VU: Vulnerable, LC: Least Concern).

Species (IUCN status)	Contribution to community change		Population trend
	Long-term change (Impact)	Medium-term change (Impact)	Short-term change (1997-2012)
*Emberiza rustica* (VU; Decreasing)	Decreased (Low)	Sharply decreased (High)	Stable
*Emberiza rutila* (LC; Stable)	Increased (Low)	Sharply decreased (High)	Stable
*Emberiza spodocephala* (LC; Stable)	Increased (High)	Increased (Moderate)	Stable
*Emberiza elegans* (LC; Stable)	Increased (High)	Increased (Low)	Decreasing (-1.82%/year)
*Emberiza cioides* (LC; Stable)	Sharply decreased (Low)	Decreased (Low)	Decreasing (-2.99%/year)
*Emberiza tristrami* (LC; Stable)	Increased (Low)	Increased (Low)	Decreasing (-0.48%/year)

### Population changes in buntings

**Fig 3:**
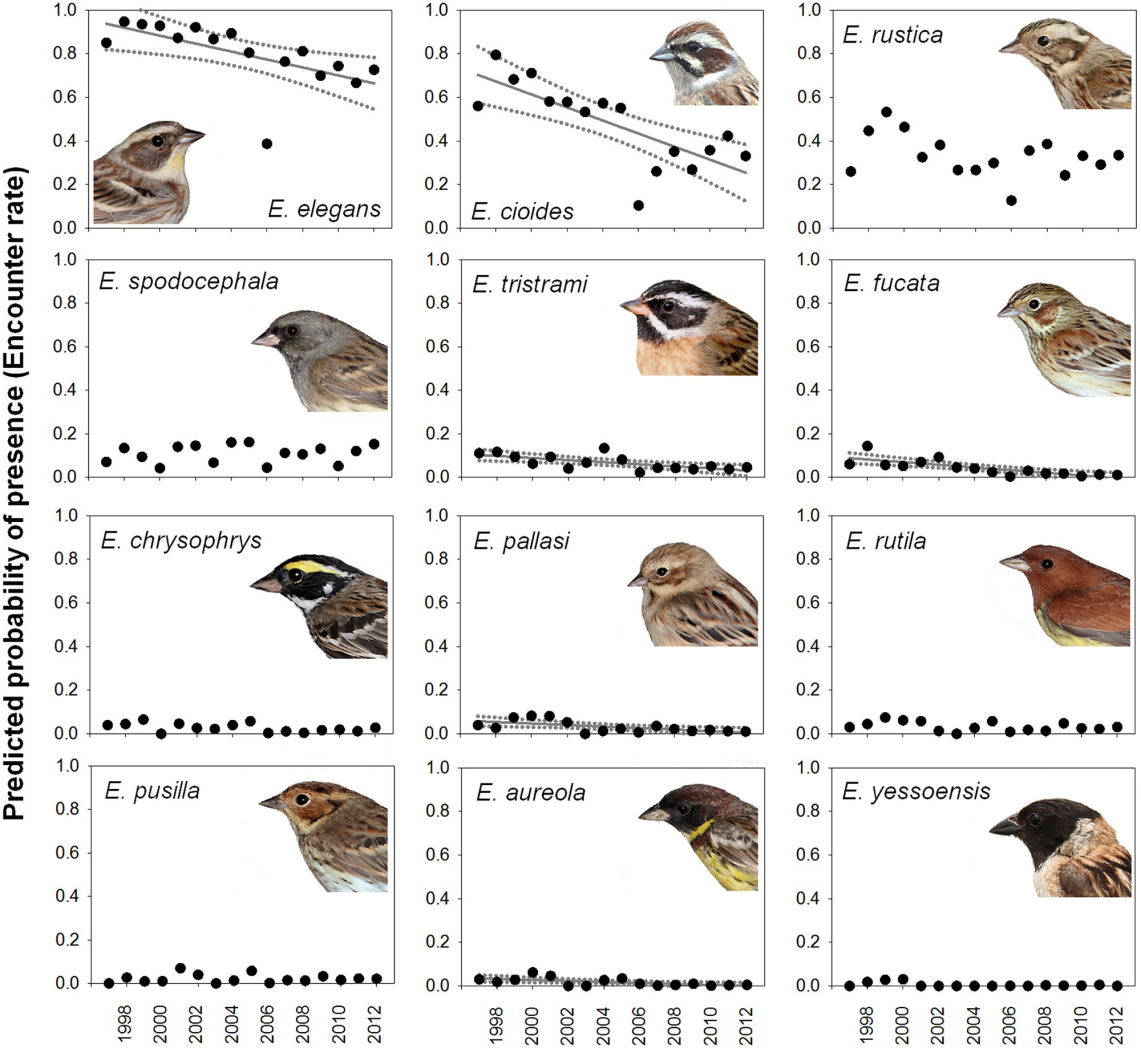
Temporal change in the predicted probability of presence (encounter rate) of *Emberiza* species in Korea. The changes between 1997 and 2012 were based on the Korean Nationwide Natural Environment Surveys. The linearly declining trend (*P* < 0.05) was found in six species: the Yellow-throated (*E. elegans*), Meadow (*E. cioides*), Tristram’s (*E. tristrami*), Pallas’s Reed (*E. pallasi*), Chestnut-eared (*E. fucata*), and Yellow-breasted Buntings (*E. aureola*). Linear regression lines (bold) and their 95% confidence lines (dotted) were given when significant.

We predicted the probability of presence for 12 species of buntings based on the presence–absence data from the NNES. The Yellow-throated and Meadow Buntings had a relatively high presence, whereas the Rustic Bunting had an intermediate presence, and other passaging and wintering buntings had a lower presence (encounter rate < 0.2) (Fig 3).

Six of the twelve bunting species showed a linearly declining trends from 1997 to 2012: Yellow-throated (slope = -0.0182, r^2^ = 0.3762, *P* = 0.012), Meadow (slope = -0.0299, r^2^ = 0.5758, *P* < 0.001), Tristram’s (*E. tristrami*; slope = -0.0048, r^2^ = 0.4572, *P* = 0.004), Pallas’s Reed (*E. pallasi*; slope = -0.0035, r^2^ = 0.3717, *P* = 0.012), Chestnut-eared (*E. fucata*; slope = -0.0061, r^2^ = 0.6113, *P* < 0.001), and Yellow-breasted Buntings (slope = -0.0023, r^2^ = 0.3464, *P* = 0.016) (Table 4). There was no known linear change in the probability of presence over time in the other species.

## Discussion

The lack of long-term data for large spatial areas has often hindered the collection of detailed knowledge of the population trends and the rates of decline of migratory songbirds in the East Asian Flyway [44]. To overcome the paucity of quantitative information, we collated specimen records and bird-banding data documented over the past 100 years for buntings, which were used as a proxy group for songbirds in Korea.

Bird banding is one of the most effective and reliable sources for information on the long-term change in bird populations as well as bird migration [20, 21]. Museum specimens are also useful materials for retrogressive studies in diverse scientific fields including taxonomy, systematics, biogeography, ecology, toxicology, evolution, and biodiversity [22, 23], and sometimes, may provide a clue on underlying mechanism of a historical change in bird populations [45]. However, unlike bird banding, collection may be a method that is less quantitative but reflects the demands of museums or collectors. Collectors are more likely to want rare species and less likely to collect more abundant species, resulting in a fairly even number of collections across species. Thus, rare species [22] or more charismatic individuals, such as showy males [23], may be selectively collected, causing a bias in relative abundance of museum collections [22]. Thus, in a species level, the abundance of a species as determined by bird banding records may not be linearly related to the number of collected specimens; this is shown in our data on Yellow-throated (395 specimens vs. 6,784 banded birds) and Black-faced Buntings (59 specimens vs. 11,092 banded birds) in Period III.

The lack of recent bird banding and ground survey data in North Korea may cause a possible bias in our results. However, the total area of the Korean Peninsula is quite small compared to the geographic ranges of the migratory buntings, and therefore, we may suggest that the difference in the status of buntings between South and North Korea is not significant in terms of the overall spatial scales of the migrants. A spatiotemporal difference in the efforts and efficiency of bird banding and collection is another inevitable factor of bias. For example, the capture rates of bird species both for banding and collection may be also affected by the diverse biotic and abiotic environments, such as geographic region, site selection, net placement, season, time of day, and vegetation cover, as well as local weather and climate conditions [46]. Nevertheless, given the quality and limitation of the available data, we did not consider the potential impacts of these subtle factors when assessing nationwide and long-term changes in this study. Instead, we interpreted the obvious changes in the species composition in a community level using data collected nationwide over a large spatiotemporal scale. Therefore, we conjectured that the bunting communities reconstructed by two independent sampling methods may be potentially biased and different in each Period. Our results revealed that the period was a more significant predictor of change in bunting communities than collection bias was, demonstrating that the relative abundances derived from museum collection data may not differ from those from bird-banding data in a community level. This does not necessarily indicate that the number of specimens independently and exactly represents the absolute abundance of each species at the time of collection. Rather, it suggests that an overall group of specimens may be a compositional index of the bird community because the more common buntings have a higher likelihood of being collected or captured in general.

Our data demonstrated that a few bunting species, such as the Meadow, Yellow-throated, Black-faced, Rustic and Chestnut Buntings which are still common or were once common, played the most important roles in the changes in bunting communities and populations over time. In particular, the long-term changes in the Korean bunting community composition between Periods I and III were largely caused by the increasing contribution of the Yellow-throated and Black-faced Buntings. However, over the past 40 years between Periods II and III, the disappearance of the Rustic and Chestnut Buntings was remarkable, while the Yellow-throated and Black-faced Buntings continued to increase their contribution. Therefore, the recent bunting communities of the 2000s have been dominated by three common species: the Yellow-throated, Meadow, and Black-faced Buntings, as also confirmed by the presence–absence data. The change in the proportion of other species in the bunting community was relatively small between the periods.

Because capturing and banding efforts have not been standardized, the numbers of banded birds between Periods II and III cannot be directly compared. Nevertheless, the considerable difference in the total number of banded buntings between the two periods (119,774 birds over 7 years in Period II vs. 29,841 birds over 27 years in Period III) may imply that there has been a sharp decline in buntings at an unknown scale. In particular, the Rustic and Chestnut Buntings, which contributed largely (58.67%) to the change in the bunting community between Periods II and III, were still ranked 3rd and 4th among bunting species banded in the 2000s; however, the drastic change in their relative abundances between Periods II and III (Rustic Bunting: from 50.43% to 8.50%; Chestnut Bunting: from 38.62% to 8.02%) raises conservation concerns for these two species.

It is known that the population of Rustic Buntings has been sharply decreasing over their entire range: there has been a 75–87% decline in overall population over the last 30 years and a 32–91% decline over the last 10 years [15]. This species is now classed as a vulnerable species (VU) in the IUCN Red List, and its population is likely decreasing [47]. However, our presence–absence data did not detect any significant change in the probability of its presence in Korea. In general, presence–absence surveys are less sensitive to changes when the population decline is modest (<20-50%) and occurs evenly over the entire sampling range [40]. Therefore, the mean encounter rate of 0.33 in this study supports the notion that the Rustic Bunting is still a common and widely-distributed wintering bunting species throughout Korea [19]. The reduced sensitivity may result in an unchanged encounter rate regardless of the suspected past and current population reduction in Korea as well as its known population collapse elsewhere [15]. However, although the Chestnut Bunting showed a very low probability of presence (mean = 0.036), there was no evidence of a population change for the species between 1997 and 2012. This species is classed as a least concern species (LC) with a stable population trend [47], but the once-common bunting is currently a uncommon migrant in Korea [19]. This finding suggests that the Chestnut Bunting had already significantly declined before 1997. Although there is no robust evidence of an ongoing population decline in Korea, its population trend in other countries should be assessed together for a better understanding of their conservational status.

In terms of short-term change, the presence–absence data showed a declining trends in six out of twelve bunting species: the Yellow-throated, Meadow, Tristram’s, Pallas’ Reed, Chestnut-eared, and Yellow-breasted Buntings. The annual change in the encounter rate or the probability of presence ranged from -0.23% for the Yellow-breasted Bunting to -2.99% for the Meadow Bunting. The Yellow-breasted Bunting, is a critically endangered species (CR) [48], has experienced a dramatic global population collapse [9, 10, 48], and it now seems to be a rare and declining species with a low probability of presence in Korea. The decline of this bunting (-0.23%/year) between 1997 and 2012 in Korea is much lower than the known global trend (decline by 84.3-94.7% between 1980 and 2013) [10, 48]. This may be because the local population passing through Korea mainly declined before 1997, or because its probability of presence was not high enough to detect the global rapid decline since 1997. For the other five species that are classified as least concern species (LC), their population trends are suspected to be stable in the absence of evidence for any decline or substantial threats [49–53]. Our results also raise new conservation concerns about the decline of these migratory bunting species.

Two common breeding bunting species in Korea, the Yellow-throated and Meadow Buntings, are described as fairly common to locally common with stable populations in their distribution ranges [49, 50]. However, both species are rapidly declining (-1.82%/year and -2.99%/year, respectively) in Korea. In particular, the Meadow Bunting seems to be the most rapidly and prominenttly declining bunting, though its contribution to the overall changes in the bunting community has not been high (3.87%) between Periods II and III. More than 3,000 individuals were banded between 1964 and 1970 (ranked 3rd in abundance) in Period II, but only 73 birds were banded and 17 specimens were collected in Period III. Its declining trend seems to be sufficiently rapid to approach the thresholds to classify it as a vulnerable species (VU) under the Red List population trend criterion (30% decline over ten years or three generations) [54]. This study does not provide any cause of this decline in the two species, but suggests that breeding buntings are under unknown but strong pressure inside Korea, such as the loss or degradation of breeding habitats due to rapid urbanization and land cover changes [55].

It is noteworthy that the Yellow-throated Bunting population is declining while at the same time its contribution to the overall bunting community composition is increasing. This suggests that bunting populations are becoming small while species diversity is simultaneously being lost in the remaining bunting communities. Although the capture rate and survey effort are directly uncomparable, the proportion of buntings to all birds captured in Korea dropped from 64% in Period II (between 1964 and 1970) [16] to 27% in Period III (between 1993 and 2017) [30], providing other evidence supporting the idea of a crisis in the buntings in Korea.

## Conclusions

Even small proportional declines in the abundance of widespread, abundant, and common species may result in large losses of individuals, biomass, and occurrences disrupting the ecosystems’ structure, function and services [56]. Furthermore, the depletion of populations of common species may have been often underestimated and overlooked [57]. Our findings raise significant concerns about the long-, medium-, and short-term decline of buntings, which are the major songbird groups in Korea. Although there have not been recent changes in the encounter rate of Rustic and Chestnut Buntings in Korea, they appear to have caused medium-term changes in bunting communities. The Chestnut Bunting has probably suffered a population collapse between the 1970s and the 1990s, and the still common Rustic Bunting may experience an undetectable modest decline. In particular, six of the twelve bunting species examined, including two common breeding species, seem to have experienced a recent decline. The increased proportion of the Yellow-throated Buntings in the long-term, despite their recent and ongoing population decline also suggests that bunting diversity is deteriorating at the same time as bunting populations are shrinking in Korea. Along with significant threats to buntings and other migratory birds such as overexploitation (illegal hunting and trade) [7, 10], habitat loss is often considered the greatest threat [1, 3]. In the Korean Peninsula, there has been total deforestation, overharvesting, pollution, and a rapid urbanization and reforestation over the past century [55, 58]. However, there is no assessment of how habitats of buntings have changed and which threats have affected buntings in Korea. Further quantitative and long-term monitoring schemes across the whole of their distribution ranges, international cooperation for identifying major threats and key areas of conservation, and law enforcement against habitat loss and illegal overexploitation are strongly required to mitigate the on-going decline in *Emberiza* buntings and migratory songbirds both in Korea and Asia.

## Supporting information

S1 Table The number of Korean *Emberiza* specimens deposited (a) in foreign countries and (b) in South and North Korea.
(PDF)

S2 Table The proportion of collected and banded *Emberiza* buntings in three different periods in Korea. (PDF)

S3 Table The number of *Emberiza* buntings captured and banded in the Period II and III in South Korea. (PDF)

S4 Table Pairwise comparison of *Emberiza* buntings communities in museum collections and bird banding data in Korea. (PDF)
