# Supplementary material for: Changes in Emberiza bunting communities and populations spanning 100 years in Korea
Source: PLoS One. 2020 May 27;15(5):e0233121. doi: 10.1371/journal.pone.0233121 (PMC7252627; doi:10.1371/journal.pone.0233121)
Supplement: S1 Table — The number of Korean Emberiza specimens deposited (a) in foreign countries and (b) in South and North Korea. (PDF) [file pone.0233121.s001.pdf]

Changes in *Emberiza* bunting communities and populations spanning 100 years in Korea

Chang-Yong Choi[1,2], Hyun-Young Nam[1,3*], Han-Kyu Kim[4¤], Se-Young Park[1], Jong-Gil Park[1]

S1 Table. The number of Korean *Emberiza* specimens deposited (a) in foreign countries and (b) in South and North Korea. Only specimens with known collection dates are included.

Species	Period I				Period II				Period III			Total
	1910s	1920s	1930s	1940s	1950s	1960s	1970s	1980s	1990s	2000s	2010s	
**(a) Foreign countries**												
Emberiza aureola	7	10	4	0	1	89	22	0	0	0	0	133
E. chrysophrys	0	7	2	0	0	0	3	0	0	0	0	12
E. cioides	6	63	20	23	81	56	18	0	0	0	0	267
E. elegans	5	27	12	18	35	41	53	2	3	2	0	198
E. fucata	4	13	7	5	17	16	25	0	0	0	0	87
E. jankowskii	0	6	0	0	0	0	0	0	0	0	0	6
E. leucocephalos	0	1	0	0	0	1	0	0	0	0	0	2
E. pallasi	1	12	5	12	10	12	7	0	0	0	0	59
E. pusilla	0	8	3	0	1	0	0	0	0	0	0	12
E. rustica	2	15	4	43	65	66	16	1	0	1	0	213
E. rutila	1	11	5	0	16	31	20	0	0	4	0	88
E. schoeniclus	1	0	0	0	0	7	10	0	0	0	0	18
E. spodocephala	21	37	5	10	17	65	25	3	0	5	0	188

E. sulphurata	0	0	0	0	0	0	0	0	0	0	0	0
E. tristrami	1	12	7	2	17	34	33	3	0	3	0	112
E. variabilis	0	0	0	0	0	0	0	0	0	0	0	0
E. yessoensis	0	9	0	0	1	7	0	0	0	0	0	17
Subtotal	49	231	74	113	261	425	232	9	3	15	0	1,412
**(b) S. & N. Korea**												
Emberiza aureola	6	0	0	0	2	12	5	6	0	4	1	36
E. chrysophrys	0	0	0	0	1	3	2	2	0	1	17	26
E. cioides	11	0	0	0	46	42	25	16	1	2	14	157
E. elegans	2	0	0	0	27	47	59	91	10	219	161	616
E. fucata	8	0	0	0	8	32	1	6	0	3	2	60
E. jankowskii	0	0	0	0	0	0	0	0	0	0	0	0
E. leucocephalos	0	0	0	0	0	9	1	0	0	0	0	10
E. pallasi	0	0	0	0	2	8	13	4	0	8	1	36
E. pusilla	0	0	0	0	4	9	0	3	0	5	3	24
E. rustica	4	0	0	0	39	85	20	44	0	12	8	212
E. rutila	5	0	0	0	10	54	43	18	2	7	13	152
E. schoeniclus	2	0	0	0	1	2	1	1	1	0	0	8
E. spodocephala	14	0	0	0	7	33	42	46	9	27	18	196
E. sulphurata	0	0	0	0	0	1	0	0	2	1	6	10
E. tristrami	2	0	0	0	12	13	20	34	8	15	35	139
E. variabilis	0	0	0	0	0	0	0	0	1	0	8	9
E. yessoensis	2	0	0	0	2	15	0	0	0	1	32	52
Subtotal	56	0	0	0	161	365	232	271	34	305	319	1,743
**Total**	105	231	74	113	422	790	464	280	37	320	319	3,155
