# Supplementary material for: Changes in Emberiza bunting communities and populations spanning 100 years in Korea
Source: PLoS One. 2020 May 27;15(5):e0233121. doi: 10.1371/journal.pone.0233121 (PMC7252627; doi:10.1371/journal.pone.0233121)
Supplement: S2 Table — (PDF) [file pone.0233121.s002.pdf]

Changes in *Emberiza* bunting communities and populations spanning 100 years in Korea

Chang-Yong Choi[1,2], Hyun-Young Nam[1,3*], Han-Kyu Kim[4¤], Se-Young Park[1], Jong-Gil Park[1]

S2 Table. The proportion of collected and banded *Emberiza* buntings in three different periods in Korea. Raw data were given in Table 1.

Species	Proportion of specimens collected (%)			Proportion of birds banded (%)	
	**Period I (1910s-1940s)**	**Period II (1950s-1980s)**	**Period III (1990s-2010s)**	**Period II (1950s-1980s)**	**Period III (1990s-2010s)**
Emberiza aureola	5.16	7.00	0.74	0.22	0.75
E. chrysophrys	1.72	0.56	2.66	0.02	4.78
E. cioides	23.52	14.52	2.51	2.63	0.24
E. elegans	12.24	18.15	58.43	2.57	22.73
E. fucata	7.07	5.37	0.74	0.74	0.92
E. jankowskii	1.15	0.00	0.00	0.00	0.00
E. leucocephalos	0.19	0.56	0.00	0.01	0.02
E. pallasi	5.74	2.86	1.33	0.00	3.29
E. pusilla	2.10	0.87	1.18	0.05	4.76
E. rustica	13.00	17.18	3.11	50.98	8.62
E. rutila	4.21	9.82	3.85	39.10	8.12
E. schoeniclus	0.57	1.12	0.15	0.01	0.89
E. spodocephala	16.63	12.17	8.73	1.65	37.17
E. sulphurata	0.00	0.05	1.33	0.01	0.43
E. tristrami	4.59	8.49	9.02	1.74	7.11
E. variabilis	0.00	0.00	1.33	0.00	0.05
E. yessoensis	2.10	1.28	4.88	0.29	0.12
**Total**	100	100	100	100	100
