# Supplementary material for: Changes in Emberiza bunting communities and populations spanning 100 years in Korea
Source: PLoS One. 2020 May 27;15(5):e0233121. doi: 10.1371/journal.pone.0233121 (PMC7252627; doi:10.1371/journal.pone.0233121)
Supplement: S3 Table — (PDF) [file pone.0233121.s003.pdf]

Changes in *Emberiza* bunting communities and populations spanning 100 years in Korea

Chang-Yong Choi[1,2], Hyun-Young Nam[1,3*], Han-Kyu Kim[4¤], Se-Young Park[1], Jong-Gil Park[1]

S3 Table. **The number of *Emberiza* buntings captured and banded in the Period II and III in Korea**. There was no organized bird banding activities prior to the 1960s.

Species	Period II	Period III			Total
	1964-1970	1993-2017	2017-2019	Subtotal	
Emberiza aureola	266	224	0	224	490
E. chrysophrys	18	1,396	30	1,426	1,444
E. cioides	3,156	73	0	73	3,229
E. elegans	3,074	6,709	75	6,784	9,858
E. fucata	888	274	1	275	1,163
E. jankowskii	0	0	0	0	0
E. leucocephalos	9	5	1	6	15
E. pallasi	0	907	76	983	983
E. pusilla	57	1,336	85	1,421	1,478
E. rustica	61,055	2,539	33	2,572	63,627
E. rutila	46,826	2,409	13	2,422	49,248
E. schoeniclus	11	265	0	265	276
E. spodocephala	1,972	10,897	195	11,092	13,064
E. sulphurata	11	127	0	127	138
E. tristrami	2,079	2,089	33	2,122	4,201
E. variabilis	0	14	0	14	14
E. yessoensis	352	34	1	35	387
**Total**	119,774	29,298	543	29,841	149,615
