# Supplementary material for: Changes in Emberiza bunting communities and populations spanning 100 years in Korea
Source: PLoS One. 2020 May 27;15(5):e0233121. doi: 10.1371/journal.pone.0233121 (PMC7252627; doi:10.1371/journal.pone.0233121)
Supplement: S4 Table — (PDF) [file pone.0233121.s004.pdf]

Changes in *Emberiza* bunting communities and populations spanning 100 years in Korea

Chang-Yong Choi[1,2], Hyun-Young Nam[1,3*], Han-Kyu Kim[4¤], Se-Young Park[1], Jong-Gil Park[1]

S4 Table. **The pairwise comparison of *Emberiza* buntings communities in museum collections and bird banding data in Korea.** There was no organized bird banding activities prior to the 1960s. The combined data were given in Table 3.

**Species**	**Abundance (%)**		**Average contribution (%)**	**Cumulative contribution (%)**
	**Earlier**	**Later**		
**Period I vs. III (Collection)**				
Emberiza elegans	12.38	58.43	27.61	48.04
Emberiza cioides	23.79	2.51	8.84	63.43
Emberiza rustica	13.15	3.11	3.92	70.25
Emberiza tristrami	4.64	9.02	3.09	75.62
Emberiza fucata	7.16	0.74	2.67	80.26
Emberiza spodocephala	16.83	8.73	2.34	84.33
**Period II vs. III (Collection)**				
Emberiza rustica	17.18	3.11	12.00	22.12
Emberiza cioides	14.52	2.51	10.14	40.87
Emberiza spodocephala	12.17	8.73	6.80	53.44
Emberiza rutila	9.82	3.85	6.31	65.10
Emberiza aureola	7.00	0.74	5.02	74.37
Emberiza tristrami	8.49	9.02	3.99	81.74
**Period II vs. III (Banding)**				
Emberiza rustica	50.98	8.62	39.09	47.18
Emberiza rutila	39.10	8.12	29.68	83.00
Emberiza spodocephala	1.65	37.17	6.07	90.36
Emberiza elegans	2.57	22.73	2.48	93.35
Emberiza cioides	2.63	0.24	2.06	95.84
Emberiza chrysophrys	0.02	4.78	0.94	96.98
